# The Workhouse Prisoner

**Published:** 1905-04-08

**Authors:** 


### THE WORKHOUSE PRISONER.

### A POOR-LAW PROBLEM.

The heading, "Workhouse Mutiny: Not Allowed to Look for Work," in a newspaper of recent date, will probably arouse both surprise and sympathy among the public, who do not realise how complicated are the problems with which the Poor-law has to deal. This is one of the most difficult. A man, driven by misfortune into the workhouse with his wife and children, must either remain there—for the rest of his life if he chooses—or take all his family out with him when he leaves. An honest man very reasonably will feel that this law handicaps him severely in his search for work. He cannot drag wife and children about the streets with him from one workshop to another while he makes his request, and when night comes where is he to house them? Yet he may be refused the permission to go out alone, even though he promises to take out his family when he gets work. Thus stated, the case is undeniably hard. But against that must be set the Guardians' side of the matter. Almost every union has its record of men, thus trusted to go out and look for work, who have disappeared from the neighbourhood, and if they have ever been heard of more, it has been after the police have been set to work to discover them, and they are charged with deserting their wives and children. It is the opposition of these two points of view which brought ten men before Mr. Rose, the Southwark magistrate, recently, charged with refusing to perform the tasks allotted to them in the workhouse. The defence was that this refusal was meant only as a means of bringing before the court, and ultimately before the public, the fact that the mutinous paupers had been refused permission to go out without their wives and children to look for work. The clerk to the Guardians admitted that the men had asked the visiting committee to allow them to go out, and had been refused, but pointed out that some of these men had already been prosecuted for deserting their wives and families, whereby these latter became chargeable to the union. It is entirely within the discretion of the Guardians whether or not they accede to such requests; there is no law nor even any uniform rule in the matter. In some unions men are allowed out once or twice a week to look for work, while in others the men are allowed to go out for a fortnight on end, on condition that they report themselves at the end of that time. If they faithfully fulfil this condition their leave may be extended for a fortnight longer, but if at the end of that time they have not found work, the inference is that their prospects are bad, and they are compelled to return to the workhouse or take out their families. If a man who had once been allowed to go out and had failed to report himself were to ask for leave to go out on a subsequent occasion, he would certainly be refused, for obvious reasons. The long list of deserters whose names, with the promised but seldom-claimed reward for capturing them, hang outside every workhouse, shows clearly that the Guardians cannot permit every man who asks leave to do so to get outside their jurisdiction, unless they want the ratepayers to be saddled for years with the cost of maintaining his family. It is the costly experience of the past that makes Poor-law officials so often turn a deaf ear to requests such as that of the Southwark mutineers. Yet at a time like this, when the slackness of trade has undeniably driven into the workhouse many men who never needed relief before, it would be well to interpret the law with as much flexibility as possible. One man told the magistrate that he absolutely had the offer of work, but would not be allowed to go out and start unless he at once took out his wife and six children with him. He wanted a week or so to earn enough to take a lodging for them before he removed them from the workhouse. It was unfortunate for this man's plea that he had already been charged with deserting his family, a fact which could not fail to prejudice the officials of the workhouse, and through them the Guardians, against him. Yet it is a pity to keep him at the expense of the ratepayers if, to vary the words of the song, "he wants to work, and is willing to work, and can get work to do." In such a case would it be possible to allow him to go out daily to work and return to the workhouse in the evening, and at the end of a week, when he had received his first pay, be discharged with his whole family? That such a privilege might be abused may be admitted, but the present system is clearly not perfect, and in a time when fresh experiments in Poor-law administration are in the air this modest one might be worth trying.

